# The effect of remote ischemic conditioning on mortality after kidney transplantation: the systematic review and meta-analysis of randomized controlled trials

**DOI:** 10.1186/s13643-024-02618-w

**Published:** 2024-07-29

**Authors:** Eunji Ko, Ha Yeon Park, Choon Hak Lim, Hyun Jung Kim, Yookyung Jang, Hyunyoung Seong, Yun Hee Kim, Hyeon Ju Shin

**Affiliations:** 1 Department of Anesthesiology and Pain Medicine, Korea University Anam Hospital, 73, Goryeodae-Ro, Seongbuk-Gu, Seoul, 02841 South Korea; 2 Department of Anesthesiology and Pain Medicine, College of Medicine, Korea University, 73, Goryeodae-Ro, Seongbuk-Gu, Seoul, 02841 South Korea; 3 Department of Preventive Medicine, College of Medicine, Korea University, 73, Goryeodae-Ro, Seongbuk-Gu, Seoul, 02841 South Korea; 4 Department of Anesthesiology and Pain Medicine, Changwon Hanmaeum Hospital, 21, Woni-Daero 682Beon-Gil, Seongsan-Gu, Changwon-Si, 51497 South Korea

**Keywords:** Delayed graft function, Kidney transplantation, Mortality, Rejection, Remote ischemic conditioning

## Abstract

**Background:**

Ischemic-reperfusion injury resulting from kidney transplantation declines the post-transplant graft function. Remote ischemic conditioning (RIC) is known to be able to reduce the criticality of ischemic reperfusion injury. This study aimed to meta-analyze whether the application of remote ischemic conditioning to kidney transplantation patients improves clinical outcomes.

**Methods:**

Researchers included randomized controlled studies of the application of RIC to either kidney donors or recipients. Articles were retrieved from PubMed, Embase, Web of Science, and Cochrane Library. The risk of bias was evaluated using RoB 2.0. The primary outcome was mortality after transplantation. Secondary outcomes were the incidence of delayed graft function, graft rejection, and post-transplant laboratory results. All outcomes were integrated by RevMan 5.4.1.

**Results:**

Out of 90 papers, 10 articles (8 studies, 1977 patients) were suitable for inclusion criteria. Mortality collected at all time points did not show a significant difference between the groups. Three-month mortality (RR, 3.11; 95% CI, 0.13–75.51, *P* = 0.49) tended to increase in the RIC group, but 12-month (RR, 0.70; 95% CI, 0.14–3.45, *P* = 0.67) or final-reported mortality (RR, 0.49; 95% CI, 0.23–1.06, *P* = 0.07) was higher in the sham group than the RIC group. There was no significant difference between the RIC and sham group in delayed graft function (RR, 0.64; 95% CI, 0.30–1.35, *P* = 0.24), graft rejection (RR, 1.13; 95% CI, 0.73–1.73, *P* = 0.59), and the rate of time required for a 50% reduction in baseline serum creatinine concentration of less than 24 h (RR, 0.98; 95% CI, 0.61–1.56, *P* = 0.93).

**Conclusions:**

It could not be concluded that the application of RIC is beneficial to kidney transplantation patients. However, it is noteworthy that long-term mortality tended to decrease in the RIC group. Since there were many limitations due to the small number of included articles, researchers hope that large-scale randomized controlled trials will be included in the future.

**Systematic review registration:**

PROSPERO CRD42022336565.

**Supplementary Information:**

The online version contains supplementary material available at 10.1186/s13643-024-02618-w.

## Background

Kidney transplantation (KT) is the most frequently performed organ transplantation surgery worldwide [1]. In 2020, out of about 130,000 solid organ transplants worldwide, 81,000 cases, or 62%, were KT. KT is currently the final treatment for patients with end-stage renal disease, and it is showing a high success rate due to its rapid development [2].

If rejection and dysfunction occur after KT, graft survival is reduced [3] It is known that the ischemic-reperfusion injury (IRI) that occurs during KT has a major influence on the deterioration of kidney graft function [4]. Therefore, many strategies are being tried to reduce KT’s IRI. Remote ischemic conditioning (RIC) is a method of performing temporary compression and reperfusion of the remote limb not near the critical organ. Since Murry's experimental study [5], RIC has been studied extensively as a method of attenuating ischemic reperfusion injury. Its effectiveness has been demonstrated in many critical IRI situations including cerebral infarction and myocardial infarction [6, 7].

Because RIC is believed to be effective for IRI, there are studies that have applied RIC to KT accompanying critical IRI [8, 9]. Although there were cases where the reduction of estimated glomerular filtration rate (eGFR) after KT was more effective in the group to which conditioning was applied [9, 10], the other study showed no significant difference in the reduction of creatinine[8]. there is still some controversy about the beneficial effects of RIC.

Although similar systematic reviews or meta-analyses have already been conducted [10, 11], the time has passed, and researchers planned to re-analyze with a focus on mortality and graft function as a clinical outcome. Therefore, researchers aimed to collect randomized controlled trials (RCTs) of patients undergoing kidney transplantation who underwent remote ischemic conditioning and comprehensively review the effect of RIC. In particular, researchers decided to focus on mortality after KT among many clinical prognoses. After living donor KT in the USA, the recipient’s 1-year survival rate reached 98.8% in 2019, but the 5-year survival rate was 87.1% [12]. Our study tried to find out whether RIC can help improve the posttransplant survival rate, which has stagnated since 2018 despite many advances in posttransplant management.

## Methods

### Design

This review was written based on the PRISMA statement (Additional file 1). The analysis method was performed in the same way as registered in PROSPERO (PROSPERO 2022 CRD42022336565). Original outcomes notified to PROSPERO included length of hospital stay and duration or rate of intensive care unit admission. However, since most studies did not report them, it was omitted that it would not be possible to conduct an acceptable analysis. In addition, it was changed after discussion that mortality represented a clinical outcome rather than tCr50, which was thought to be the primary outcome. PICO is as follows:

- P: Patients underwent KT
- I: Applying RIC during the perioperative period
- C: Sham group (patients who were not applied RIC)
- O: Mortality (at posttransplant 3 and 12 months, at the period when finally reported), graft function (incidence of delayed graft function [DGF], rejection within 12 months after transplantation), laboratory results (eGFR at 12 months after transplantation, the time required to a 50% decrease in baseline serum creatinine concentration [tCr50])

All prospective randomized controlled studies in which adult and pediatric patients receiving KT were divided into RIC-applied group and control sham group and compared clinical outcomes were eligible. In addition to full-text articles, abstracts, letter to editor, and brief communication prior to publication were included if the trials were randomized controlled. No limitation on language, countries, and publication year was applied. Articles in which only genetic or molecular outcomes were identified were excluded. Studies that did not apply conditioning to the remote limbs but only applied local ischemic conditioning were also excluded.

Studies were last retrieved on September 5, 2022, from the PubMed, EMBASE, Web of Science, and Cochrane libraries. The search strategy can be checked in Additional file 2. With the help of an information specialist, a search strategy was developed with reference to previous systematic reviews and RCTs. Searching terms included “Remote Ischemic Conditioning” and “kidney transplantation”. In addition, additional articles were collected by referring to the citations. Last systematic reviews, related articles, and ongoing studies from clinical trials (clinicalTrials.gov) were searched initially to prevent omissions.

Two researchers (E.K. and H.Y.P.) searched the databases and judged inclusion in the abstract. Afterwards, articles were retrieved and the inclusion was determined. All steps were performed in an independent and consistent manner. Finally, one of the corresponding authors (Y.H.K.) decided on the included studies.

#### Data collection

Data were extracted from the included studies. Two investigators (E.K. and H.Y.P.) independently performed the procedure. One of the corresponding authors (H.J.S.) lastly checked the data for errors.

#### Assessment of risk of bias

The risk of bias in all papers was evaluated based on the Cochrane tool for assessing the risk of bias in randomized trials (RoB 2 tool)[13]. The RoB 2 tool assessed (1) bias arising from the randomization process, (2) bias due to deviation from the intended intervention, (3) bias due to missing outcome data, (4) bias in outcome measurement, and (5) bias in the selection of reported outcomes, respectively, and consequently assessed the overall risk of bias. The risk of bias was independently evaluated by 2 researchers (E.K. and H.Y.P.) and then reviewed by one corresponding author (Y.H.K.). The effect of interest was the effect of assignment to the interventions at baseline, which was estimated by an intention-to-treat analysis.

#### Assessment of quality of evidence

The quality of evidence for each outcome was assessed with the “Grading of Recommendations Assessment, Development, and Evaluation (GRADE)” [14]. The GRADE system classifies the quality of evidence into one of four levels: high, medium, low, and very low. This evaluation is the result of five reasons: (1) study limitations, (2) inconsistency of results, (3) indirectness of evidence, (4) imprecision of results, and (5) risk of bias. The results of the RoB assessment have influenced the quality of evidence.

#### Outcome measures

The primary outcome was mortality. The mortality which were reported within posttransplant 3 and 12 months, or reported the latest period of each study were collected. As secondary outcomes, the incidence of delayed graft function and rejection were confirmed as variables related to kidney graft function. To evaluate changes in postoperative blood tests, tCr50 and eGFR at 12 months after transplantation were collected. DGF was defined as hemodialysis within 1 week. The results from articles that confirmed DGF by definitions other than the one mentioned were not integrated. Graft rejection within 12 months after transplantation included biopsy-proven rejection according to the Banff criteria [15], clinical acute rejection, or steroid-resistant rejection.

The types of articles included, nationality, number of patients, and methods of RIC were collected. Depending on the manner in which RIC was performed, the outcomes were collected whether it was applied to the donor or recipient, how many cycles were performed, and whether the application was pre-, per-, or post-conditioning. Remote ischemic preconditioning (RIPreC) or preconditioning (RIPerC) means performing conditioning before or during a critical ischemic event of the target organ (kidney graft) occurs, respectively. Patients underwent remote ischemic postconditioning (RIPostC) induced after ischemia of the target organ, at the initiation of reperfusion.

#### Synthesis method

Among the outcomes extracted from the included articles, the outcomes researchers want to analyze in this review were selected and synthesized by using RevMan 5.4.1 (Cochrane Collaboration, Oxford, UK). Outcomes were evaluated through the risk ratio (RR) for categorical outcomes and the mean difference (MD) for continuous variables under random effect. The results expressed in the median and interquartile range (IQR) were converted to mean and standard deviation (SD) [16]. After converting to mean and SD, rounding was performed to one decimal place. eGFR can be evaluated in various ways, such as the equation of chronic kidney disease epidemiology collaboration (CKD-EPI equation) or modification of diet in renal disease (MDRD equation). Since the evaluation methods were different, the standard mean difference (SMD) was evaluated. If multiple articles were published for one study, the authors of RCTs were contacted by mail for advice so that the appropriate one of the duplicated results could be selected. When the outcome to be extracted was not recorded in the articles, researchers mailed the author of the trial to check once more. The primary analysis included outcomes from all eligible studies regardless of risk of bias assessments.

Since there is no standardized RIC strategy applied to KT patients yet, there were some differences in the method of applying RIC in each study. Therefore, researchers tried to offset the heterogeneity between studies with a random effect model rather than a fixed effect model. The degree of heterogeneity among studies was expressed by *I*^2^ statistics and the ranges of 0–50%, 50–75%, and 75–100% were regarded as low, moderate, and high heterogeneity, respectively. For outcomes with high heterogeneity, subgroup analysis was performed to examine which factors were related to heterogeneity. According to the initiation time of conditioning (pre-, per-, or post-conditioning), the number of limb compressions applied during conditioning (3 or 4 cycles), and whether conditioning was performed on KT donors or recipients were classified.

Sensitivity analysis was performed by evaluating outcomes which include the studies with low risk of bias. Publication bias was evaluated by funnel plots when the outcomes of at least 10 studies were available to synthesize.

## Results

### Study characteristics

On September 5th, 2022, 18, 30, and 42 articles were retrieved from the three databases of PubMed, EMBASE, Web of Science, and Cochrane Library, respectively. Of the total of 90 articles, 34 were duplicated records, and 17 articles that were only registered in the study protocol or ongoing clinical trials were excluded. Remained 39 articles were screened and 24 were excluded by confirming the abstract. Fifteen articles were retrieved and 10 were assessed for eligibility (Table 1). One abstract that was difficult to determine whether it is randomized controlled [17] and four abstracts that can be replaced with full-text articles were excluded. In addition to database searching, 3 articles were found as a result of the search through the citation of the previous studies, and one (MacAllister’s full-text article of the REPAIR study) could be added by excluding two that were not RCT. However, the abstract of the REPAIR study [18] was excluded as a duplicate of the newly included full-text article. Among the studies, Nielsen’s article was a 1-year follow-up report of Krogstrup’s CONTEXT study, and Veighey’s article was a long-term outcome of MacAllister’s REPAIR study. Finally, 8 studies and 10 reports were included. This can be confirmed in the PRISMA 2020 flow diagram in Additional file 3. Table 1 summarizes the characteristics of the included articles. From this paragraph on, all included studies were not mentioned in citation lists.

**Table 1:** Characteristics of included studies

1st author	Year	Nation	Design	Donor organ	Sample size (intervention/control)	Interventions			Primary outcome of recipient
						Type	Site	Cycle, compression time	Secondary outcome of recipient
Bang	2019	South Korea	RCT	Living donor	Donors 170 (85/85) Recipients 168 (85/83)	Preconditioning to donor	Upper arm	3, 5 min	• Serum creatinine level (sCr) at discharge • Estimated glomerular function rate (eGFR) at discharge
									• Time required for a 50% decrease in baseline sCr concentration (tCr50) • eGFR at postoperative 12 months • Incidence of delayed graft function (DGF) • Acute rejection within postoperative 12 months • Graft failure within postoperative 12 months
Bongu	2017	USA	RCT (poster only)	Brain death donor	320 (155/166)	Preconditioning to donor	Thigh	4, 5 min	• Incidence of DGF • Death-censored kidney graft survival
Chen	2013	China	RCT (Letter to Editor)	Living donor	60 (20/20/20)	Preconditioning to donor or recipient	Upper leg	3, 5 min	• Incidence of DGF • Urine volume within postoperative 72 h • sCr within postoperative 72 h • Plasma neutrophil gelatinase-associated lipocalin (NGAL) within postoperative 72 h • Urine retinol-binding protein (RBP) within postoperative 72 h • Urine N-acetyl-D-glucosaminidase (NAG) activity within postoperative 72 h • Plasma superoxide dismutase (SOD) activity within postoperative 72 h • Plasma malondialdehyde (MDA) within postoperative 72 h • Postoperative stay in hospital (PODs) • Total costs during hospitalization • Severe adverse effects
Kim	2014	South Korea	RCT, Double-blinded	Living donor	60 (30/30)	Postconditioning to recipient	Upper arm, free of AV fistula	3, 5 min	• sCr during postoperative 96 h • eGFR during postoperative 96 h • Daily urine output until postoperative day 7 • Daily urine creatinine until postoperative day 7 • Incidence of acute rejection • incidence of DGF • Length of hospital stay (LOS) • POD • tCr50 • Incidence of tCr50 less than 12, 24, and 36 h • Incidence of sCr normalization time less than 48, 72, 96 h • sCr at postoperative 1 year • eGFR at postoperative 1 year
Krogstrup (CONTEXT study)	2017	Denmark, Sweden, Netherlands (4 centers, 3 nations)	RCT	Brain/cardiac death donor	222 (109/113)	Preconditioning to recipient	Thigh, contralateral to transplant side	4, 5 min	• tCr50
									• Primary non-function (PNF) • Postoperative dialysis • Measured GFR(mGFR) at postoperative day 5 • eGFR at postoperative day 21 • Plasma NGAL during postoperative 3 days • LOS • Serious adverse events within postoperative 7 days
MacAllister (REPAIR study)	2015	UK, Netherlands, Belgium, France (13 centers, 4 nations)	RCT, double-blinded	Living donor	391 (98/99/99/95)	Preconditioning to donor and recipient	Upper arm	4, 5 min	• mGFR at postoperative 12 months
									• tCr50 • eGFR at postoperative 3, 12 months • Plasma interleukin 6 (IL-6), IL-1beta, interferon-gamma (IFN-gamma), tumor necrosis factor-alpha (TNF-alpha) during postoperative 1 ~ 5 days • Protein expression changes in renal tissue • Renal graft cortical tubulointerstitial fibrosis at postoperative 6 months • Incidence of DGF • Incidence of acute rejection within postoperative 12 months • sCr and eGFR at postoperative 2 to 5 years • Patient survival at postoperative 12 months and 2 to 5 years • Graft survival at postoperative 3 months and 2 to 5 years
Nicholson	2015	UK	RCT, double-blinded	Living donor	80 (40/40)	Preconditioning to recipient	Thigh, contralateral to transplant site	4, 5 min	• eGFR at postoperative 1 and 3 months
									• sCr at postoperative 1 and 3 months • Incidence of PNF • Incidence of DGF • Creatinine reduction ratio (CRR) at postoperative day 2 • Incidence of acute rejection • Incidence of graft failure
Nielsen	2019	Denmark, Sweden, Netherlands (4 centers, 3 nations)	RCT, 1 year follow-up of CONTEXT study	Brain/cardiac death donor	222 (109/113)	Preconditioning to recipient	Thigh, contralateral to transplant side	4, 5 min	• tCr50
									• mGFR at postoperative at postoperative 3 and 12 months • eGFR at postoperative 3 and 12 months • sCr, plasma cystatin C, urine Cr, urine albumin at postoperative 3 and 12 months • Plasma NGAL at postoperative 3 and 12 months • Incidence of DGF • Incidence of rejection within postoperative 3 and 12 months • Incidence of new-onset diabetes after transplantation within postoperative 3 and 12 months • Patient survival within postoperative 3 and 12 months
Veighey	2019	UK, Netherlands, Belgium, France (13 centers, 4 nations)	RCT, Long-term follow-up of REPAIR study	Living donor	406 (102/103/102/99)	Preconditioning to donor and recipient	Upper arm	4, 5 min	• Changes of eGFR during postoperative 5 years • Graft loss within postoperative 5 years • Mortality at postoperative 3 months • Annual mortality within postoperative 5 years
Wu	2014	China	RCT	Cardiac death donor	48 (24/24)	Preconditioning to recipient	Unilateral lower limb by clamping the external iliac artery	3, 5 min	• Incidence of DGF • Incidence of acute rejection • eGFR during postoperative 30 days • Urine NGAL • Renal tissue analysis

Mortality, which is the primary endpoint, was confirmed in 4 articles (MacAllister, Nicholson, Nielsen, Veighey). Among the outcomes to evaluate kidney graft function, DGF was extracted in 5 articles (Kim, Krogstrup, MacAllister, Nicholson, Wu), and graft rejection was confirmed in 3 articles (Bang, MacAllister, Nielsen). Among the laboratory results, 3 articles reported tCr50 as a continuous variable (Bang, Kim, Krogstrup), which other 3 articles recorded the number of patients with tCr50 achieved within 24 h (Kim, MacAllister, Nicholson). eGFR at 12 months after transplantation was extracted the results from 4 articles (Bang, Kim, MacAllister, Nielsen).

**Fig. 1:**
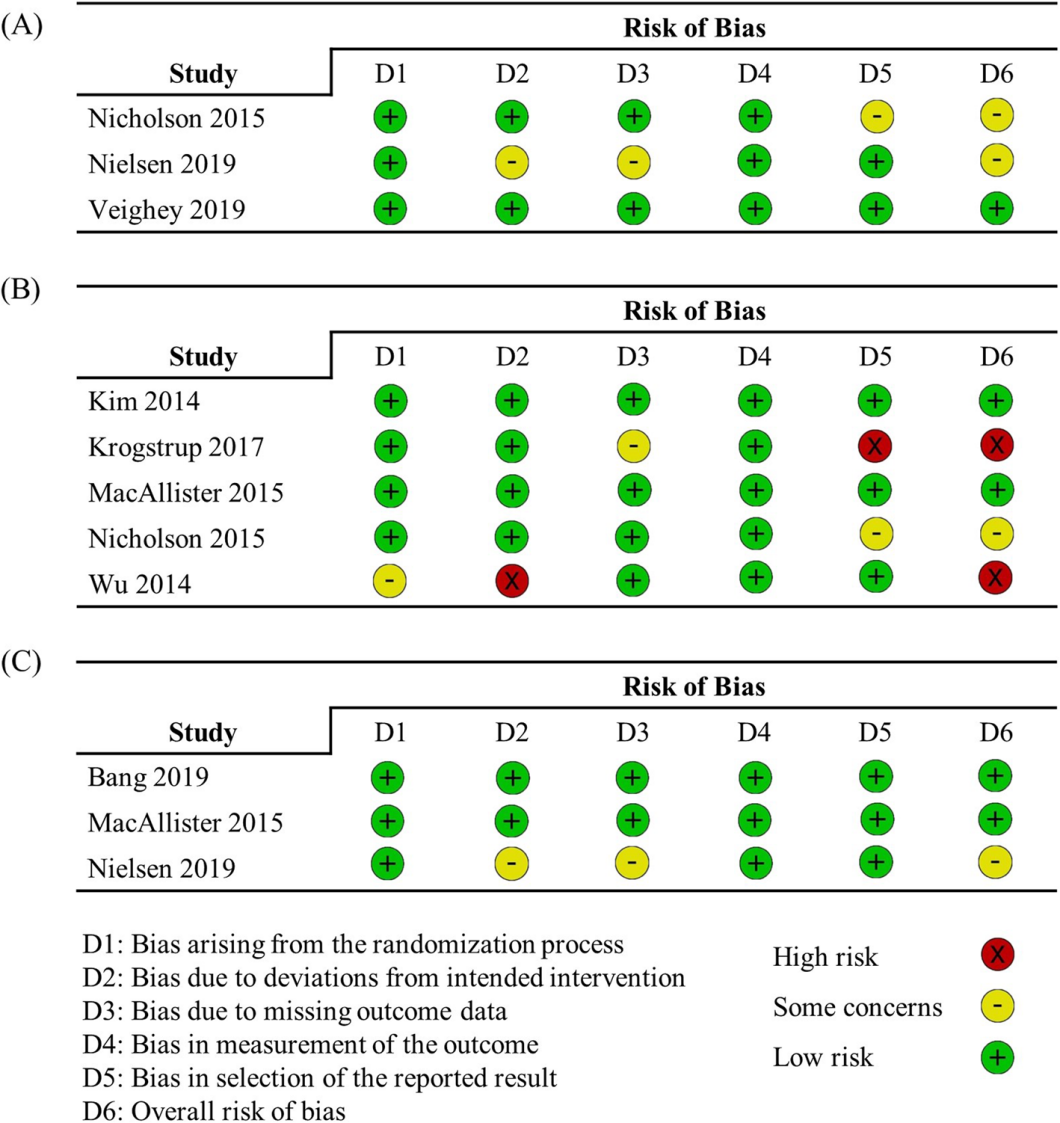
Assessment of the risk of bias by the Cochrane risk of bias tool (RoB 2). RoB was evaluated for three outcomes: **A** the mortality finally reported, **B** the incidence of delayed graft function, and **C** graft rejection within 12 months posttransplant

The risk of bias results finally determined by the corresponding author are shown in Fig. 1. Figure 1A is the risk of bias for finally-reported mortality, which is the primary outcome. Figure 1B, C shows a risk of bias assessments for secondary outcomes, delayed graft function, and acute rejection, respectively. The risk of bias evaluated by two independent researchers showed an 80% concordance rate, and None of the risk of bias assessments reported contradictory results, with one researcher assigning a low risk while the other assigning a high risk for the same item. Two studies that were not full-text articles did not evaluate the risk of bias (Bongu, Chen). Two out of 8 full-text articles were at high risk (Krogstrup, Wu). Four were low risk of bias (Bang, Kim, MacAllister, Veighey). Publication bias could not be evaluated because of few included studies.

### Effect of RIC

#### Primary endpoint (Fig. 2)

**Fig. 2:**
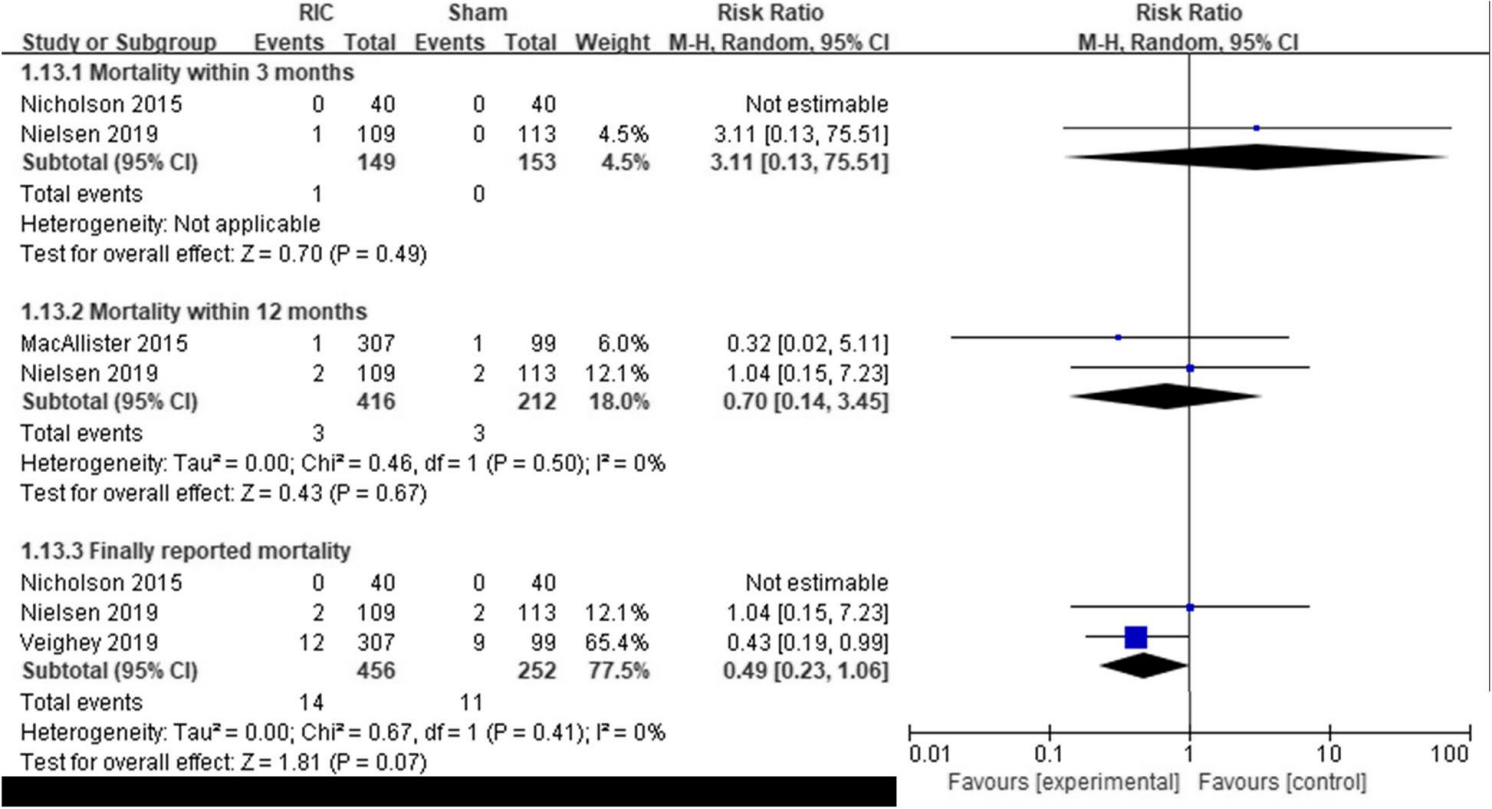
Forest plots evaluating the primary outcomes of remote ischemic conditioning for patients who underwent kidney transplantation. Each plot shows the effect on mortality at 3 and 12 months after transplantation and finally reported mortality

Mortality of patients within 3 months of transplantation was reported in 302 patients in two studies (Nicholson, Nielsen). However, since no death occurred in Nicholson's study, it could not be synthesized into the outcome. 1 out of 249 in the RIC group and 0 out of 153 in the sham group died within 3 months, and there was no significant difference in mortality (RR, 3.11; 95% CI, 0.13–75.51, *P* = 0.49).

Mortality of patients within 12 months of transplantation was reported in 628 patients in two studies (MacAllister, Nielsen). Three out of 416 patients in the RIC group and 3 out of 212 in the Sham group died, and the mortality rates were not significantly different (RR, 0.70; 95% CI, 0.14–3.45, *P* = 0.67). Heterogeneity was low (*I*^2^ = 0%).

The mortality finally reported in the included studies could be found in four articles (MacAllister, Nicholson, Nielsen, Veighey). Since the follow-up study of MacAllister’s report was Veighey’s report, only Veighey’s results were used. Fourteen out of 456 in the RIC group and 11 out of 252 in the sham group died. There was no significant difference between the two groups, but clinically, the mortality rate was reduced by half in the RIC group (RR, 0.49; 95% CI, 0.21–1.11, *P* = 0.09). Heterogeneity was low (*I*^2^ = 0%).

Among the mortality rates collected at the three time periods, short-term mortality (3-month post-transplant mortality) tended to increase in the RIC group, but mid- to long-term mortality (12-month post-transplant, or the latest reported mortality) was higher in the sham group than in the RIC group.

#### Secondary endpoint (Fig. 3)

**Fig. 3:**
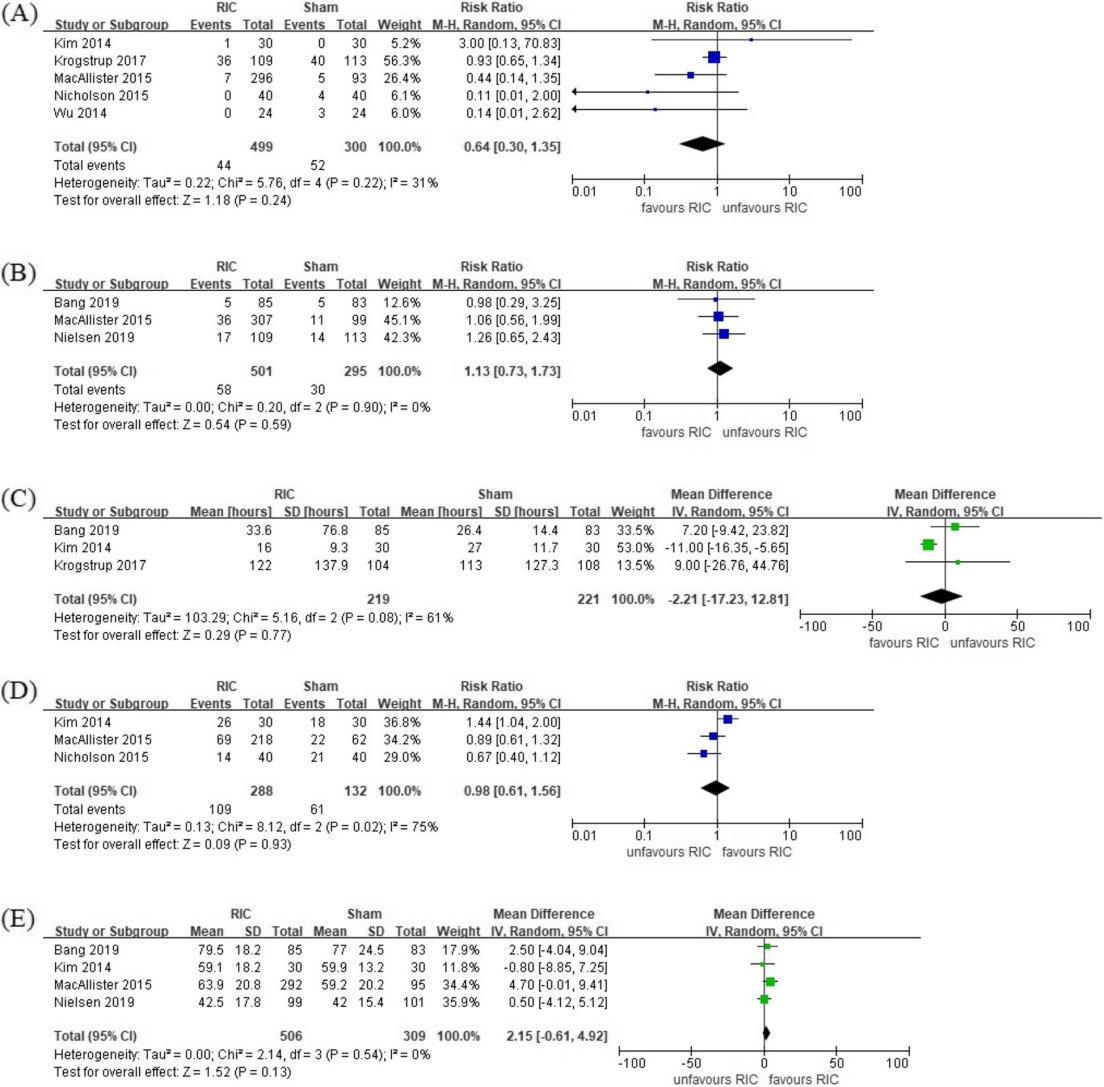
Forest plots evaluating the secondary outcomes of remote ischemic conditioning for patients who underwent kidney transplantation. Each plot shows the incidence of delayed graft function (A), the incidence of graft rejection within 12 months (B), the time required for a 50% decrease in baseline serum creatinine concentration [tCr50, hours] (C), the incidence of tCr50 less than 24 h (D), and eGFR at postoperative 12 months [ml/minute/1.73 m.^2^] (E)

DGF occurred in 103 of a total of 1027 patients. It occurred in 50 out of 624 patients in the RIC group and 53 out of 403 in the sham group. Although the incidence was less in the RIC group, there was no significant difference (RR, 0.64; 95% CI, 0.30–1.35, *P* = 0.24). It showed low heterogeneity (*I*^2^ = 31%). Graft rejection within posttransplant 12 months occurred in 88 of 796 patients. There were 58 of 501 in the RIC group and 30 of 295 in the sham group, and there was no difference between the two groups (RR, 1.13; 95% CI, 0.73–1.73, *P* = 0.59) (Fig. 3B). Heterogeneity was very low.

Of the 440 patients in whom tCr50 could be confirmed, it took 29.0 h for 219 patients in the RIC group and 26.6 h for 221 patients in the sham group. Again, there was no difference between the two groups (MD − 2.21 h; 95% CI − 17.23–12.81, *P* = 0.77). Heterogeneity was moderate (*I*^2^ = 61%). The number of patients with tCr50 within 24 h was 170 out of 420 patients. One hundred nine of 288 patients in the RIC group and 61 of 132 in the sham group were within 24 h, and there was no significant difference between the two groups (RR, 0.98; 95% CI, 0.61–1.56, *P* = 0.93). Heterogeneity was almost high (*I*^2^ = 75%). The eGFR at 12 months after transplantation was 62.0 and 58.4 ml/minute/1.73 m^2^ in RIC-applied 506 patients and 309 patients in the sham group, respectively. The eGFRs of the two groups were not significantly different (MD 2.15 ml/minute/1.73 m^2^; 95% CI − 0.61–4.92, *P* = 0.13). SMD was 0.12 (95% CI − 0.02–0.27, *P* = 0.10). The heterogeneity was very low (*I*^2^ = 0%).

All outcomes that excluded non-low-risk studies were re-evaluated for sensitivity analysis. The validity of the 3-month mortality rate could not be assessed as it did not include any low-risk studies. Both mortality within 12 months (RR, 0.32; 95% CI, 0.02–5.11, *P* = 0.42) and finally reported mortality (RR, 0.43; 0.19–0.99, *P* = 0.05) remained non-significant differences between the RIC group and sham group. Secondary outcomes, including DGF (RR, 0.64; 95% CI, 0.14–2.94, *P* = 0.57), graft rejection (RR, 1.04; 95% CI, 0.59–1.82, *P* = 0.90), tCr50 (MD − 3.67 h; 95% CI − 21.17–13.82, *P* = 0.68), tCr50 within 24 h (RR, 1.15; 95% CI, 0.69–1.90, *P* = 0.59), and eGFR (3.07–0.38) − 6.53 0.08) all yielded non-significant results. A funnel plot to evaluate publication bias could not be created because there was no integrated result of more than 10 studies across all outcomes. Additional file 4 provides the quality of each result value evaluated using the GRADE. All primary outcomes were found to lack precision due to the small number of studies and participants. In addition, studies related to tCr50 did not always show the same tendency and showed high heterogeneity, so the inconsistency was evaluated as serious. Because the RIC protocol was slightly different for each study, serious indirectness was evaluated in terms of indirectness when the location or time period of the application of RIC was different. As a result, mortality within 12 months, the incidence of delayed graft function, and tCr50 were judged to be of very low-quality evidence. The other outcomes also appear to be low quality of evidence.

#### Subgroup analysis

Although a plan for subgroup analysis was registered in PROSPERO, the authors judged that the number of studies included in this meta-analysis was so small that a subgroup analysis with sufficient evidence could not be performed. Therefore, the results of the subgroup analysis were not reported.

## Discussion

This systematic review and meta-analysis is the latest review that analyzed how clinical outcomes change when RIC is performed on KT patients. Although it has been known that RIC can help the prognosis of patients in the pathological condition accompanying IRI, the results of this meta-analysis showed that RIC did not have a significant benefit on the mortality and graft function of KT recipients. In addition, since the number of studies applying RIC to KT patients is still small (*n* = 8), subgroups affecting clinical outcomes have not been identified.

Looking at previous reviews, in Farooqui’s paper, solid organ transplant recipients (human or animal) with RIPreC were included [11]. Five studies on people who performed KT were included, but studies that performed RIPostC were omitted because they were limited to RIPreC. It was also different from our study in that the statistical significance could not be confirmed because a meta-analysis was not performed. Zhou’s study in 2017 had a very similar topic to this study, but it was published 5 years ago and the number of included trials was limited to six [10]. The most recent paper to conduct a meta-analysis on a similar topic is Zhang’s study [19]. The implementation of RIC showed a significant but weak effect in reducing serum creatinine and improving eGFR and did not prove a significant effect on other outcomes. However, the analysis excluded some RCTs (Bongu, Krogstrup, MacAllister, Veighey, Wu) and focused on the effect on laboratory results rather than clinical outcomes such as mortality. The current review was able to target more patients with the addition of 4 articles since Zhou’s study (Bang, Bongu, Nielsen, Veighey). Ultimately, this systemic review and meta-analysis is meaningful in that it comprehensively covers the effects of RIC applied to the latest KT patients.

Mortality at posttransplant 12 months and DGF were likely to be lower in the RIC group than in the sham group, and the RIC group had less tCr50 and higher eGFR than the sham group. However, in the case of early mortality and graft rejection, the sham group was smaller, and the number of patients who achieved tCr50 within 24 h was more in the sham group. Therefore, it was difficult to say that the positive tendency of all outcomes was inclined toward the RIC group.

This study focused on mortality in the prognosis of KT with RIC. After KT, the mortality rate of recipients has decreased due to the development of immunotherapy, but the risk of dying from cardiovascular disease or infection remains [20]. Ying’s analysis showed that despite a dramatic decrease in mortality after initial transplantation, the first 3 months are still a period of high risk of death from cardiovascular disease and infections, while cancer and cardiovascular disease are the leading causes of death after 1 year after KT [21]. Of course, as age develops, the mortality rate tends to decrease, but the initial mortality rate is still high [22]. In our study, mortality was classified and confirmed for 3 months and 12 months, and it was found that mortality decreased in the RIC group in studies that collected the data of mortality later (Fig. 2). It is difficult to expect that RIC reduced the short-term mortality through this meta-analysis so far. Indeed, the evidence is still lacking as only two studies confirmed mortality at 3 months after transplantation. Researchers further need RCT for these objectives; first, RCT to examine the effect of RIC among KT on short-term mortality after KT. Second, RCT to determine whether the application of RIC among KTs is associated with cardiovascular disease or cancer in the long term.

In order to perform meta-analysis, it was necessary to collect the processed results in a unified way, but there were differences between studies. Especially, the definition of DGF varied from study to study [23]. There were 7 articles that confirmed DGF as an outcome, but 2 were omitted because the definitions were not the same. There were some articles that measured the change in the level of eGFR or serum creatinine, but it was difficult to synthesize because the measurement time and unit were different. Although continuous observation is usually performed on 1 day, 7 days, 3 months, 6 months, and 1 year after transplantation, there is a lack of research related to the most meaningful blood study conducted at any time. For the development of research about KT, it was thought that it was necessary to have a unified definition of outcomes identified in the study.

The biggest limitation of this review is the small number of included papers. The publication bias could not be confirmed due to the small number of papers. Later, through re-review in the future, researchers will synthesize the outcome of increasing studies and evaluate the publication bias again. Another limitation was that subgroup analysis was not possible due to the small number of papers.

## Conclusions

KT studies are worthy of large-scale RCTs. It is the most frequently performed transplantation and the success rate of transplantation continues to rise. Although there is a multicenter trial in which RCT is applied to KT patients, if such a large-scale study is added with a standardized protocol, it will be possible to end the debate about the effectiveness of RIC. In particular, although significant results have not yet been obtained in this meta-analysis, it is hoped that studies on whether it can increase the mid- to long-term survival rate of transplant recipients will be published.

### Supplementary Information

Additional file 1. PRISMA checklist.

Additional file 2. Search strategy.

Additional file 3. Flow Chart for identification of included studies. This flow diagram is based on PRISMA 2020 flow diagram for new systematic reviews.

Additional file 4. Quality of evidence. CI = confidence interval; MD = mean difference; RR = risk ratio.

## Data Availability

The datasets used and/or analyzed during the current study are available from the corresponding author on reasonable request.

## Abbreviations

CKD-EPI equation: The equation of chronic kidney disease epidemiology collaboration
DGF: Delayed graft function
eGFR: Estimated glomerular filtration rate
GRADE: Grading of recommendations assessment, development, and evaluation
IQR: Interquartile range
IRI: Ischemic reperfusion injury
KT: Kidney transplantation
MD: Mean difference
MDRD equation: Modification of diet in renal disease
RCTs: Randomized controlled trials
RIC: Remote ischemic conditioning
RIPerC: Remote ischemic preconditioning
RIPostC: Remote ischemic postconditioning
RIPreC: Remote ischemic preconditioning
RR: Risk ratio
SD: Standard deviation
SMD: Standard mean difference
tCr50: Time required to a 50% decrease in baseline serum creatinine concentration
