# Supplementary material for: The effect of remote ischemic conditioning on mortality after kidney transplantation: the systematic review and meta-analysis of randomized controlled trials
Source: Syst Rev. 2024 Jul 29;13:201. doi: 10.1186/s13643-024-02618-w (PMC11285121; doi:10.1186/s13643-024-02618-w)
Supplement: Supplementary file 2 — Additional file 2. Search strategy. [file 13643_2024_2618_MOESM2_ESM.docx]

Additional file 2. Search strategy

| **Database** | **Search strategy** |
| --- | --- |
| MEDLINE | 1. “Kidney Transplantation” [MeSH Terms] 2. "Kidney Transplantation" [TIAB] OR "Renal Transplantation" [TIAB] OR "Kidney Transplant" [TIAB] OR "Renal Transplant" [TIAB] 3. 1 OR 2 4. “Ischemic Postconditioning” [MeSH Terms] OR “Ischemic Preconditioning” [MeSH Terms] 5. "remote ischemic" [TIAB] OR "remote ischaemic" [TIAB] OR "limb ischemic" [TIAB] OR "limb ischaemic" [TIAB] 6. RIC[TIAB] OR RLIC[TIAB] OR RIPC[TIAB] OR RIPreC[TIAB] OR RIPrC[TIAB] OR "rIPeC" [TIAB] OR "RIPerC" [TIAB] OR "RPerC" [TIAB] OR "RIPoC" [TIAB] OR "RIPostC" [TIAB] 7. 4 OR 5 OR 6 8. 3 AND 7 9. 8 AND (groups[TIAB] OR trial[TIAB] OR randomly[TIAB] OR placebo[TIAB] OR randomized[TIAB] OR "controlled clinical trial"[Publication Type] OR "randomized controlled trial"[Publication Type]) 10. 9 NOT (animals[MeSH Terms] NOT (humans[MeSH Terms] AND animals[MeSH Terms])) 11. 10 NOT ("review"[Publication Type] OR “Editorial”[Publication Type] OR “Meta-Analysis”[Publication Type] OR “Case Reports”[Publication Type] OR "review literature as topic"[MeSH Terms] OR “Meta-Analysis as Topic”[MeSH Terms]) |
| EMBASE | 1. 'kidney transplantation'/exp 2. 'kidney transplantation':ab,ti OR 'kidney transplant':ab,ti OR 'renal transplantation':ab,ti OR 'renal transplant':ab,ti 3. #1 OR #2 4. 'remote ischemic conditioning'/exp 5. 'remote ischemic':ab,ti OR 'remote ischaemic':ab,ti OR 'limb ischemic':ab,ti OR 'limb ischaemic':ab,ti 6. ric:ab,ti OR rlic:ab,ti OR ripc:ab,ti OR riprec:ab,ti OR riprc:ab,ti OR ripec:ab,ti OR riperc:ab,ti OR rperc:ab,ti OR ripoc:ab,ti OR ripostc:ab,ti 7. #4 OR #5 OR #6 8. #3 AND #7 9. #8 AND ('crossover procedure'/exp OR 'double blind procedure'/exp OR 'randomized controlled trial'/exp OR 'single blind procedure'/exp OR random* OR factorial* OR crossover* OR 'cross over' OR 'cross-over' OR placebo* OR (doubl* AND blind*) OR (singl* AND blind*) OR assign* OR allocat* OR volunteer*) 10. #9 NOT ('animal cell'/de OR 'animal experiment'/de OR 'animal model'/de OR 'animal tissue'/de OR 'meta analysis'/de OR 'meta analysis (topic)'/de OR 'nonhuman'/de OR 'systematic review'/de) 11. #10 NOT ('conference review'/it OR 'review'/it) |
| Cochrane | 1. MeSH descriptor: [Kidney Transplantation] explode all trees 2. (kidney transplantation):ti,ab,kw OR (kidney transplant):ti,ab,kw OR (renal transplantation):ti,ab,kw OR (renal transplant):ti,ab,kw 3. #1 OR #2 4. MeSH descriptor: [Ischemic Preconditioning] explode all trees 5. MeSH descriptor: [Ischemic Postconditioning] explode all trees 6. (remote ischemic):ti,ab,kw OR (remote ischaemic):ti,ab,kw OR (limb ischemic):ti,ab,kw OR (limb ischaemic):ti,ab,kw 7. #4 OR #5 OR #6 8. #3 AND #7 in Trials |
