# Supplementary material for: The effect of remote ischemic conditioning on mortality after kidney transplantation: the systematic review and meta-analysis of randomized controlled trials
Source: Syst Rev. 2024 Jul 29;13:201. doi: 10.1186/s13643-024-02618-w (PMC11285121; doi:10.1186/s13643-024-02618-w)
Supplement: Supplementary file 3 — Additional file 3. Flow Chart for identification of included studies. This flow diagram is based on PRISMA 2020 flow diagram for new systematic reviews. [file 13643_2024_2618_MOESM3_ESM.docx]

Additional file 3. Flow Chart for identification of included studies. This flow diagram is based on PRISMA 2020 flow diagram for new systematic reviews.

**Identification of studies via other methods**

**Identification of studies via databases and registers**

Records removed *before screening*:

Duplicate records removed (n = 34)

Records marked as ineligible by automation tools (n = 0)

Records removed for other reasons (n = 17; protocols reported at clinical trials)

Records identified from:

Websites (n = 0)

Organisations (n = 0)

Citation searching (n = 3)

etc.

Records identified from*:

Databases (n = 3)

Registers (n = 90)

**Identification**

Records screened

(n = 39)

Records excluded**

(n = 24)

Reports not retrieved

(n = 0)

Reports sought for retrieval

(n = 3)

Reports sought for retrieval

(n = 15)

Reports not retrieved

(n = 0)

**Screening**

Reports excluded:

Reason 1 (n = 2) Not RCT

Reports assessed for eligibility

(n = 1)

Reports assessed for eligibility

(n = 10)

Reports excluded:

Reason 1 (n = 4) only abstract and full text available to replace

Reason 2 (n = 1) abstract that cannot be judged as RCT

Reports excluded:

Reason 1 (n = 1) only abstract and full text available to replace

Studies included in review

(n = 8)

Reports of included studies

(n = 10)

**Included**
