# Supplementary material for: The effect of remote ischemic conditioning on mortality after kidney transplantation: the systematic review and meta-analysis of randomized controlled trials
Source: Syst Rev. 2024 Jul 29;13:201. doi: 10.1186/s13643-024-02618-w (PMC11285121; doi:10.1186/s13643-024-02618-w)
Supplement: Supplementary file 4 — Additional file 4. Quality of evidence. CI = confidence interval; MD = mean difference; RR = risk ratio. [file 13643_2024_2618_MOESM4_ESM.docx]

Additional file 4. Quality of evidence. CI = confidence interval; MD = mean difference; RR = risk ratio

| **Certainty assessment** | | | | | | | **№ of patients** | | **Effect** | | **Certainty** | **Importance** |
| --- | --- | --- | --- | --- | --- | --- | --- | --- | --- | --- | --- | --- |
| **№ of studies** | **Study design** | **Risk of bias** | **Inconsistency** | **Indirectness** | **Imprecision** | **Other considerations** | **KT RIC** | **placebo** | **Relative (95% CI)** | **Absolute (95% CI)** |  |  |
| **Mortality within 3 months** | | | | | | | | | | | | |
| 2 | randomised trials | not serious | not serious^a^ | serious^b^ | very serious^c,d^ | strong association | 1/149 (0.7%) | 0/153 (0.0%) | **RR 3.11** (0.13 to 75.51) | **0 fewer per 1,000** (from 0 fewer to 0 fewer) | ⨁⨁◯◯ Low |  |
| **Mortality within 12 months** | | | | | | | | | | | | |
| 2 | randomised trials | not serious | not serious^a^ | very serious^b,e^ | serious^d^ | none | 3/416 (0.7%) | 3/212 (1.4%) | **RR 0.70** (0.14 to 3.45) | **4 fewer per 1,000** (from 12 fewer to 35 more) | ⨁◯◯◯ Very low |  |
| **Mortality, finally-reported** | | | | | | | | | | | | |
| 3 | randomised trials | not serious | not serious^a^ | very serious^b,e,f^ | serious^d^ | strong association | 14/456 (3.1%) | 11/252 (4.4%) | **RR 0.49** (0.23 to 1.06) | **22 fewer per 1,000** (from 34 fewer to 3 more) | ⨁⨁◯◯ Low |  |
| **Incidence of Delayed Graft Function** | | | | | | | | | | | | |
| 5 | randomised trials | serious | not serious^a^ | very serious^b,e^ | not serious | none | 44/499 (8.8%) | 52/300 (17.3%) | **RR 0.64** (0.30 to 1.35) | **62 fewer per 1,000** (from 121 fewer to 61 more) | ⨁◯◯◯ Very low |  |
| **(Acute) Rejection within 12 months** | | | | | | | | | | | | |
| 3 | randomised trials | not serious | not serious^a^ | very serious^b,e^ | not serious | none | 58/501 (11.6%) | 30/295 (10.2%) | **RR 1.13** (0.73 to 1.73) | **13 more per 1,000** (from 27 fewer to 74 more) | ⨁⨁◯◯ Low |  |
| **tCr50** | | | | | | | | | | | | |
| 3 | randomised trials | serious | serious^a,g^ | very serious^b,e^ | not serious^h^ | none | 219 | 221 | - | MD **2.21 lower** (17.23 lower to 12.81 higher) | ⨁◯◯◯ Very low |  |
| **tCr50 <24hr** | | | | | | | | | | | | |
| 3 | randomised trials | not serious | serious^a,g^ | serious^e^ | not serious^h^ | none | 109/288 (37.8%) | 61/132 (46.2%) | **RR 0.98** (0.61 to 1.56) | **9 fewer per 1,000** (from 180 fewer to 259 more) | ⨁⨁◯◯ Low |  |
| **eGFR at 12 months** | | | | | | | | | | | | |
| 4 | randomised trials | not serious | not serious^a^ | very serious^b,e^ | not serious^h^ | none | 506 | 309 | - | MD **2.15 higher** (0.61 lower to 4.92 higher) | ⨁⨁◯◯ Low |  |

#### Explanations

a. Unexplained variability or heterogeneity among studies

b. Differences between the population studied (age, gender or clinical status differences)

c. Small sample size to analyze

d. Only few events to analyze

e. Differences between the intervention (similar but not identical)

f. Differences between the outcomes (mixture of short-term and long-term results)

g. Not low heterogeneity

h. Broad CI
